# F122. CLINICAL CHARACTERISTICS OF LATE-ONSET SCHIZOPHRENIA IN COMPARISON WITH EARLY-ONSET SCHIZOPHRENIA: ONE YEAR FOLLOW-UP STUDY

**DOI:** 10.1093/schbul/sby017.653

**Published:** 2018-04-01

**Authors:** Jung Suk Lee, Mi-Ae Ko, Seon-Koo Lee

**Affiliations:** 1 NHIS Ilsan Hospital; 2 Bundang Jesaeng Hospital

## Abstract

**Background:**

Late-onset schizophrenia (LOS) differs from early-onset schizophrenia (EOS) in several ways including predominance of women, better premorbid social adjustment and lower severity of positive/negative symptoms. However, no studies evaluated the longitudinal course of LOS. This study aimed to investigate the clinical characteristics of LOS in comparison with EOS and the longitudinal course of clinical symptoms and functioning in LOS.

**Methods:**

By reviewing medical records, we assessed demographic data, clinical characteristics, and general functioning of 20 LOS (5 males) and 44 EOS (16 males) who admitted to National Health Insurance Service Ilsan Hospital. LOS and EOS were defined according to age at first onset: ≥40 years (LOS) and <40 years (EOS). The level of clinical symptoms were rated using the Positive and Negative Syndrome Scale (PANSS), and general functioning was evaluated using the General Assessment of Functioning (GAF).

**Results:**

There was no significant difference in gender between LOS and EOS. The mean ages of onset were 45.4 ± 3.97 (LOS) and 28.4 ± 6.69 (EOS) years. Significantly more LOS patients (90.0%) had a marital history including divorce than EOS (56.8%). There were no differences between LOS and EOS in the positive, negative, and general scores of PANSS measured at admission and 1 year after. LOS patients had significantly higher score of PANSS N2 item (Emotional withdrawal) both at admission (LOS: 4.00 ± 1.34; EOS: 3.43 ± 1.52) and 1 year after (LOS: 3.50 ± 1.00; EOS: 2.91 ± 1.05) than EOS. There were negative correlations between GAF (1 year after) and N2 item score (at admission: r= –0.45, p=0.04; 1 year after: r= –0.85, p<0.001) in the LOS group, but no significant correlation exists in the EOS group.

**Discussion:**

Consistent with previous studies, our study suggested that LOS patients had better premorbid social functioning because marital history can be regarded as index of premorbid social adjustment. However, on the contrary to previous findings, LOS patients had more severe emotional withdrawal and it was related to worse functioning. This finding may be due to cultural specificity in Korea; thus, further studies with larger samples are needed for confirmation.

